# Journal publications by Australian chiropractic academics: are they enough?

**DOI:** 10.1186/1746-1340-14-13

**Published:** 2006-07-27

**Authors:** Wayne Hoskins, Henry Pollard, John Reggars, Andrew Vitiello, Rod Bonello

**Affiliations:** 1 PhD candidate, Macquarie Injury Management Group, Department of Health and Chiropractic, Macquarie University, Australia; 2 Senior lecturer, Macquarie Injury Management Group, Department of Health and Chiropractic, Macquarie University, Australia; 3 Private practice of chiropractic, Ringwood, Victoria, Australia; 4 Associate Professor, Macquarie Injury Management Group, Department of Health and Chiropractic, Macquarie University, Australia

## Abstract

**Purpose:**

To document the number of journal publications attributed to the academic faculty of Australian chiropractic tertiary institutions. To provide a discussion of the significance of this output and to relate this to the difficulty the profession appears to be experiencing in the uptake of evidence based healthcare outcomes and cultures.

**Methods:**

The departmental websites for the three Australian chiropractic tertiary institutions were accessed and a list of academic faculty compiled. It was noted whether each academic held a chiropractic qualification or research Doctoral (not professional) degree qualification A review of the literature was conducted using the names of the academics and cross-referencing to publications listed independently in the PubMed and Index to Chiropractic Literature (ICL) databases (from inception to February 27 2006). Publications were excluded that were duplicates, corrected reprints, conference abstracts/proceedings, books, monographs, letters to the editor/comments or editorials. Using this information an annual and recent publication rate was constructed.

**Results:**

For the 41 academics there was a total of 155 PubMed listed publications (mean 3.8, annual rate per academic 0.31) and 415 ICL listed publications (mean 10.1, annual rate 0.62). Over the last five years there have been 50 PubMed listed publications (mean 1.2, annual rate 0.24) and 97 ICL listed publications (mean 2.4, annual rate 0.47). Chiropractor academics (n = 31) had 29 PubMed listed publications (mean 2.5, annual rate 0.27) and 265 ICL listed publications (mean 8.5, annual rate 0.57). Academics with a doctoral degree (n = 13) had 134 PubMed listed publications (mean 10.3, annual rate 0.70) and 311 ICL listed publications (mean 23.9, annual rate 1.44). Academics without a Doctoral degree (n = 28) had 21 PubMed listed publications (mean 0.8, annual rate 0.13) and 104 ICL listed publications (mean 3.7, annual rate 0.24).

**Conclusion:**

While several academics have compiled an impressive list of publications, overall there is a significant paucity of published research authored by the majority of academics, with a trend for a falling recent publication rate and not having a doctoral degree being a risk factor for poor publication productivity. It is suggested that there is an urgent necessity to facilitate the acquisition of research skills in academic staff particularly in research methods and publication skills. Only when undergraduate students are exposed to an institutional environment conducive to and fostering research will concepts of evidence based healthcare really be appreciated and implemented by the profession.

## Background

The production of research by chiropractors has been extremely limited for many years [1]. This historic lack of endeavor appears to be universal. The nature of publication output of Australian academics has not yet been measured. Despite many Australian chiropractic academics having called for a commitment to research [2,3], very little actual output appears to be demonstrable by the majority of academics. This is curious given the widespread knowledge that publication is a fast track to promotion in academia [4,5]. Despite this concern, Australia has produced only the third PubMed Central indexed chiropractic journal [6]. *Chiropractic & Osteopathy*, which commenced in 2005, follows the *Journal of Manipulative Physiological Therapeutics *(*JMPT*) and *Chiropractic History *as being the only chiropractic journals to be indexed in PubMed. However, *Chiropractic & Osteopathy *is the first to have open access status as an online journal allowing articles to become freely and universally accessible online, permitting the widest possible dissemination of chiropractic research and literature. So whilst a conduit for the dissemination of research is available in this and the other chiropractic/biomedical journals, it appears that Australian chiropractic academics have, by large, not actually taken advantage of this opportunity. Why?

This scenario establishes a significant problem for the profession as a whole in that it is likely that the poor research output and poor application of new "technology"/paradigms into chiropractic, notably evidence based practice (EBP), will ultimately impede the implementation and uptake of these paradigms to effect meaningful change and growth within the profession. Consequently, chiropractors are often chastised as being unscientific quacks [7]. The etiology of these problems is complex and multi-factorial. It is proposed that, at the institutional level, there exists a fundamental lack of an intrinsic chiropractic 'research culture'. Possible reasons for these problems include a long held traditional 'philosophical' and unscientific viewpoint within sections of the profession [8-10]; an inability to critically appraise and apply new thinking to such dogmatic tenets; the profession being unwilling or unable to both recognize and accept change; and educators being unable to present the above skill set in the core teaching of chiropractors, due to a lack of knowledge, time, effectiveness or the pursuit of more traditional chiropractic tenet based beliefs and constraints (dogma) [11,12].

Chiropractic in Australia has grown to stage where there are now three public university based chiropractic institutions. These institutions, Macquarie University in Sydney, Murdoch University in Perth (who will graduate their first students in 2006) and the Royal Melbourne Institute of Technology (RMIT) University in Melbourne will now collectively graduate more chiropractors than ever. Moreover, these institutions are called home by more chiropractic academics than ever. It is unclear whether these academics possess the skill set and scholarly activity which is vital to the effectiveness of the institutions and to provide the modern education necessary in an ever-changing evidence-based health care environment [1]. These skill sets are essential in order to guarantee the future health and growth of the profession. Accordingly, it was the aim of this review to document the number of research publications accumulated by the academic faculty of the chiropractic tertiary institutions within Australia. Additionally, it was our aim to provide a focus of discussion on the significance of these findings, to relate this to the difficulty the profession appears to be experiencing in the uptake of evidence based health care outcomes and cultures and to provide potential methods to improve the current state of academic research.

## Methods

The chiropractic departmental websites for Macquarie University, Murdoch University and RMIT were accessed and a list of academic staff was compiled (date accessed February 27 2006) [13-15]. Administrative and non-academic staff were excluded from our analysis. It was noted whether each academic listed held a chiropractic qualification or research Doctoral (not professional) degree qualification. Where further information was required about an academic, which was not provided by the university websites, personal communication was attempted to resolve the omission. Using the names of the academic staff, a search of the literature was conducted using the PubMed and Index to Chiropractic Literature (ICL) databases from inception to February 27 2006 with results collated independently for each database. Search terms were the surname followed by first initial. An assenting hand search of the resultant citations captured was conducted in order to correctly identify and correlate the author with the publication. All publications collated over a career were tallied and attributed to the academics current institution affiliation, regardless of the previous affiliation listed on the publication. Articles were retrieved in electronic or hard copy format from the Macquarie University Library, Sydney University Library, Melbourne University Library or from personal library collections. Articles were included on the basis that they underwent a blinded peer reviewed process. Articles were excluded that were duplicates, corrected reprints, conference abstracts or proceedings, books, monographs, letters to the editor/comments or editorials. As not all books undergo peer review, they were excluded from our analysis.

A publication rate for each author was constructed using the year of the author's first publication on the respective database as a reference point. Publications between that point and 27^th ^February 2006 were then calculated to provide an annual publication rate. In addition a publication rate for each author over the last five years was also constructed.

In this paper the authors explore the reasons for the lack of research culture in chiropractic. It examines the general research output of Australian chiropractic academics and the issues associated with it rather than the individual performances of individual universities or academics. For this reason results are presented in the format of University A, B, C and academic 1, 2, 3, etc. for which institutions were randomly assigned to either group A, B or C and academics at each institution were randomly assigned a number.

## Results

**Figure 1:**
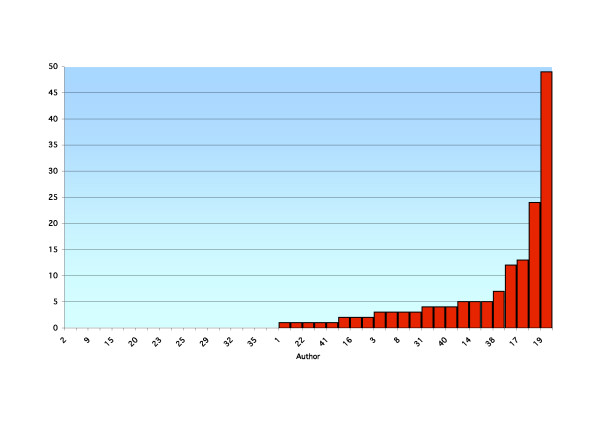
A ranked breakdown by a function of author of total publications listed in the database PubMed in February 2006.

**Figure 2:**
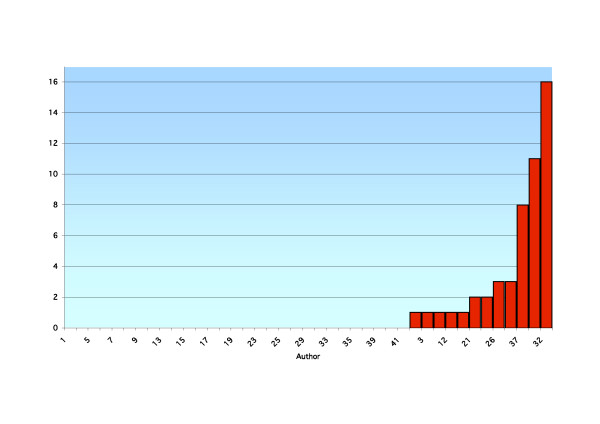
A ranked breakdown by a function of author of publications listed in the database PubMed in the last five years.

**Figure 3:**
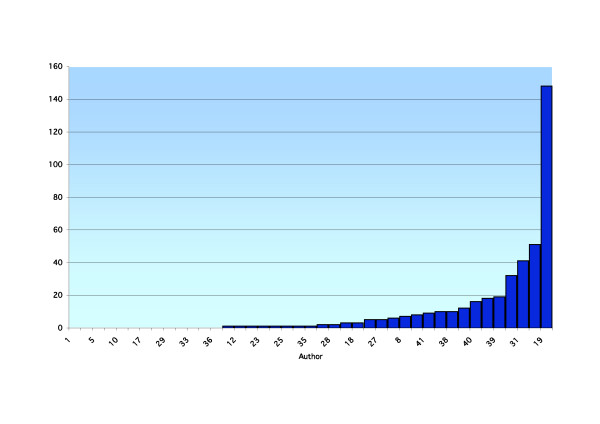
A ranked breakdown by a function of author of total publications listed in the database ICL in February 2006.

**Figure 4:**
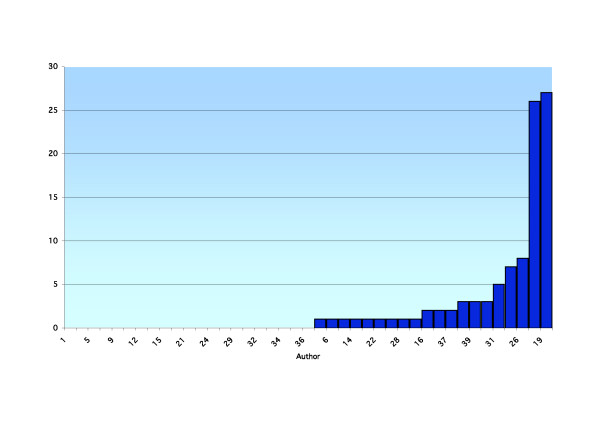
A ranked breakdown by a function of author of publications listed in the database ICL in the last five years.

Forty-one academics were reviewed using the above search criteria. Results are presented for each academic, including the total number of publications, year of first publication, year of most recent publication, annual publication rate, total number of publications over the last five years and average number of publications per year for the last five years (recent publication rate) for the PubMed (Table 1) and ICL databases (Table 2). A ranked breakdown of publications by a function of author is presented for total PubMed listed publications (Figure 1), recent PubMed listed publications (Figure 2), total ICL listed publications (Figure 3) and recent ICL listed publications (Figure 4).

**Table 1:** Australian chiropractic institution academic publications listed in the database PubMed in February 2006

Author	University	Publications	First publication	Most recent publication	Publication rate	Publications last 5 years	Recent publication rate
1*	A	1	2005	2005	1.00	1	0.20
2	A	0	-	-	0	0	0
3	A	3	1995	2005	0.27	1	0.20
4*	A	0	-	-	0	0	0
5*^	A	3	1995	1995	0.27	0	0
6^	A	1	1993	1993	0.08	0	0
7	A	2	1982	1984	0.08	0	0
8^	A	3	1998	2005	0.38	2	0.40
9	A	0	-	-	0	0	0
10*^	A	5	1991	1995	0.33	0	0
11^	A	24	1995	2006	2.18	16	3.20
12	A	0	-	-	0	0	0
13^	A	3	1995	2000	0.27	0	0
14*^	A	5	1999	2006	0.71	3	0.60
15*	A	0	-	-	0	0	0
16	B	2	2001	2001	0.40	2	0.40
17*^	B	13	1969	1989	0.35	0	0
18	B	0	-	-	0	0	0
19*^	B	49	1979	2005	1.81	11	2.20
20	B	0	-	-	0	0	0
21*	B	0	-	-	0	0	0
22	B	1	1985	1985	0.05	0	0
23*	B	0	-	-	0	0	0
24	B	0	-	-	0	0	0
25	B	0	-	-	0	0	0
26^	B	12	1997	2006	1.33	8	1.60
27	C	1	2005	2005	1.00	1	0.20
28	C	0	-	-	0	0	0
29	C	0	-	-	0	0	0
30	C	0	-	-	0	0	0
31^	C	4	1994	1999	0.33	0	0
32	C	0	-	-	0	0	0
33^	C	5	2006	1997	0.56	1	0.20
34	C	0	-	-	0	0	0
35	C	0	-	-	0	0	0
36	C	0	-	-	0	0	0
37	C	2	1988	1994	0.11	0	0
38^	C	7	1990	2004	0.44	3	0.60
39	C	4	1988	1995	0.22	0	0
40	C	4	1998	2005	0.50	1	0.20
41	C	1	1990	1990	0.06	0	0

*Denotes non-chiropractor.

^Denotes doctoral degree

**Table 2:** Australian chiropractic institution academic publications listed in the database Index to Chiropractic Literature in February 2006

Author	University	Publications	First publication	Most recent publication	Publication rate	Publications last 5 years	Recent publication rate
1*	A	0	-	-	0	0	0
2	A	10	1986	2005	0.50	7	1.40
3	A	12	1991	2005	0.80	1	0.20
4*	A	0	-	-	0	0	0
5*^	A	0	-	-	0	0	0
6^	A	3	1993	2002	0.23	1	0.20
7	A	1	1986	1986	0.05	0	0
8^	A	7	1995	2005	0.64	1	0.20
9	A	0	-	-	0	0	0
10*^	A	0	-	-	0	0	0
11^	A	51	1994	2006	4.25	26	5.20
12	A	1	1995	1995	0.09	0	0
13^	A	18	1994	2000	1.50	0	0
14*^	A	1	2004	2004	0.50	1	0.20
15*	A	0	-	-	0	0	0
16	B	5	1992	2001	0.36	2	0.40
17*^	B	0	-	-	0	0	0
18	B	3	1999	2004	0.43	1	0.20
19*^	B	148	1985	2005	7.05	27	5.40
20	B	2	2002	2003	0.50	2	0.40
21*	B	0	-	-	0	0	0
22	B	6	1985	2001	0.29	1	0.20
23*	B	1	1997	1997	0.11	0	0
24	B	1	1989	1989	0.06	0	0
25	B	1	1997	1997	0.11	0	0
26^	B	32	1985	2005	1.52	8	1.60
27	C	5	1985	2005	0.24	1	0.20
28	C	2	1988	2002	0.11	1	0.20
29	C	0	-	-	0	0	0
30	C	1	1993	1993	0.08	0	0
31^	C	41	1989	2005	2.41	5	1.00
32	C	0	-	-	0	0	0
33^	C	0	-	-	0	0	0
34	C	0	-	-	0	0	0
35	C	1	1995	1995	0.09	0	0
36	C	0	-	-	0	0	0
37	C	8	1987	2005	0.42	2	0.40
38^	C	10	1988	2004	0.56	3	0.60
39	C	19	1985	2002	0.90	3	0.60
40	C	16	1992	2005	1.14	3	0.60
41	C	9	1985	2004	0.43	1	0.20

*Denotes non-chiropractor.

^Denotes doctoral degree

Results for all academics and for the three universities are presented in Table 3. Table 4 presents the results for the chiropractor academics combined and for the three universities. Table 5 presents the results for the non-chiropractor academics combined and for the three universities. Table 6 presents the results for academics with and without a doctoral degree and for chiropractor academics with and without a doctoral degree.

**Table 3:** Analysis of institution publications listed in the database PubMed and Index to Chiropractic Literature in February 2006

	Combined academics N = 41	University A academics N = 15	University B academics N = 11	University C academics N = 15
Total PubMed indexed publications	155	50	77	28
Mean PubMed indexed publications	3.8 (SD 8.6)	3.3 (SD 6.0)	7.0 (SD 29.3)	1.9 (SD 2.3)
Mean PubMed indexed publication rate	0.31	0.37	0.36	0.21
Total PubMed indexed publications last 5 years	50	23	21	6
Mean PubMed indexed publications last 5 years	1.2 SD 3.2	1.5 SD 4.1	1.9 SD 3.9	0.4 SD 0.8
Mean PubMed indexed recent publication rate	0.24	0.31	0.38	0.08
Total ICL indexed publications	415	104	199	112
Mean ICL indexed publications	10.1 (SD 24.8)	6.9 (SD 13.4)	18.1 (SD 44.1)	7.5 (SD 11.1)
Mean ICL indexed publication rate	0.62	0.57	0.95	0.43
Total ICL indexed publications last 5 years	97	37	41	19
Mean ICL indexed publications last 5 years	2.4 (SD 5.8)	2.5 (SD 6.7)	3.7 (SD 8.1)	1.3 (SD 1.6)
Mean ICL indexed recent publication rate	0.47	0.49	0.75	0.25

**Table 4:** Analysis of chiropractor academic publications listed in the database PubMed and Index to Chiropractic Literature in February 2006

	Total chiropractor academics N = 31	University A chiropractor academics N = 9	University B chiropractor academics N = 7	University C chiropractor academics N = 15
Total PubMed indexed publications	79	36	15	28
Mean PubMed indexed publications	2.5 (SD 4.8)	4.0 (SD 7.6)	2.1 (SD 4.4)	1.87 (SD 2.3)
Mean PubMed indexed publication rate	0.27	0.36	0.25	0.21
Total PubMed indexed publications last 5 years	35	19	10	6
Mean PubMed indexed publications last 5 years	1.1 (SD 3.2)	2.1 (SD 5.3)	1.4 (SD 3.0)	0.4 (SD 0.8)
Mean PubMed indexed recent publication rate	0.23	0.42	0.29	0.08
Total ICL indexed publications	265	103	50	112
Mean ICL indexed publications	8.5 (SD 12.4)	11.4 (SD 16.0)	7.1 (SD 11.1)	7.5 (SD 11.1)
Mean ICL indexed publication rate	0.57	0.90	0.47	0.43
Total ICL indexed publications last 5 years	69	36	14	19
Mean ICL indexed publications last 5 years	2.2 (SD 4.9)	4.0 (SD 8.5)	2.0 (SD 2.8)	1.3 (SD 1.6)
Mean ICL indexed \ recent publication rate	0.45	0.80	0.40	0.25

**Table 5:** Analysis of non-chiropractor academic publications listed in the database PubMed and Index to Chiropractic Literature in February 2006

	Total non-chiropractor academics N = 10	University A non-chiropractor academics N = 6	University B non-chiropractor academics N = 4	University C non-chiropractor academics N = 0
Total PubMed indexed publications	76	14	62	-
Mean PubMed indexed publications	7.6 (SD 15.1)	2.3 (SD 2.3)	15.5 (SD 23.2)	-
Mean PubMed indexed publication rate	0.45	0.39	0.54	-
Total PubMed indexed publications last 5 years	15	4	11	-
Mean PubMed indexed publications last 5 years	1.5 (SD 3.5)	0.7 (SD 1.2)	2.8 (SD 5.5)	-
Mean PubMed indexed recent publication rate	0.30	0.13	0.55	-
Total ICL indexed publications	150	1	149	-
Mean ICL indexed publications	15.0 (SD 26.7)	0.17 (SD 0.4)	37.3 (SD 73.8)	-
Mean ICL indexed publication rate	0.77	0.08	1.79	-
Total ICL indexed publications last 5 years	28	1	27	-
Mean ICL indexed publications last 5 years	2.8 (SD 8.5)	0.2 (SD 0.4)	6.8 (SD 13.5)	-
Mean ICL indexed recent publication rate	0.56	0.03	1.35	-

**Table 6:** Analysis of doctoral degree academic publications listed in the database PubMed and Index to Chiropractic Literature in February 2006

	Academics with a doctoral degree N = 13 (31.7%)	Academics without a doctoral degree N = 28 (68.3%)	Chiropractor academics with doctoral degree N = 8 (25.8%)	Chiropractic academics without a doctoral degree N = 23 (74.2%)
Total PubMed publications	134	21	59	20
Mean PubMed publications	10.3 (SD 13.2)	0.75 (SD 1.2)	7.4 (SD 7.5)	0.9 (SD 1.3)
Mean PubMed publication rate	0.70	0.13	0.70	0.12
Total PubMed publications last 5 years	44	6	30	5
Mean PubMed publications last 5 years	3.4 (SD 5.1)	0.2 (SD 0.5)	3.8 (SD 5.6)	0.2 (SD 0.5)
Mean PubMed recent publication rate	0.68	0.04	0.75	0.04
Total ICL publications	311	104	162	110
Mean ICL publications	23.9 (SD 41.1)	3.7 (SD 5.2)	20.3 (SD 18.9)	4.6 (SD 5.4)
Mean ICL publication rate	1.44	0.24	1.39	0.30
Total ICL publications last 5 years	72	25	44	25
Mean ICL publications last 5 years	5.5 (SD 9.6)	0.9 (SD 1.5)	5.5 (SD 8.7)	1.1 (SD 1.6)
Mean ICL recent publication rate	1.11	0.18	1.10	0.22

Of the 41 academics, 13 had a doctoral degree qualification (31.7%) and 28 did not (63.8%). University A had 7 academics with a doctoral degree (46.7% of total staff), University B had 3 (27.3% of total staff) and University C also had 3 (20.0% of total staff). Of the 31 chiropractor academics, 8 had a doctoral degree (25.8%). University A had 4 of its chiropractor academics with a doctoral degree (44.4%), University B had 1 (14.3%) and University C had 3 (20.0%). Of the 10 non-chiropractor academics, 5 had a doctoral degree (50.0%). University A had 3 non-chiropractor academics with a doctoral degree (50.0%), University B had 2 (50.0%) and University C did not have non-chiropractor academics listed on their website.

## Discussion

### Main findings

Our review revealed that there is a confronting paucity of publications produced by the majority of chiropractic academics within Australia. The average publication rate per year for all academics in the PubMed database was 0.31 and in the ICL database 0.62. Over the last five years, these figures have dropped to 0.24 and 0.47 respectively (see Table 3). In 2000, the publication rate across all Australian universities was 0.8 publication points per academic (university range 0.2 – 1.67), with one publication point equaling one journal paper [16], a figure similar to other health care professions [17,18]. Of concern is that the publication rate for the chiropractic academics at the universities assessed is 2–3 times less than their respective university average [16]. Publication often begins around the early 20's, reaches a peak around the age of 40 and then declines [19]. Those starting with a greater rate early in their career generally maintain the rate throughout their research career [19]. Based on this criterion, a healthy publication rate is achieved by only a minority of chiropractic academics. This has created an imbalance in research productivity amongst the academics such that 10% of academics have produced two thirds of the PubMed listed publications and over the last five years and more strikingly, three academics have produced three quarters of the PubMed listed publications.

Not having a doctoral degree appears to be a risk factor for poor publication productivity; academics with a doctoral degree producing a 5.2 times greater publication rate than those without, which supports previous research findings [20]. This figure is even greater if we compare output of the last five years where doctoral academics have 17 times greater output. If we compare the PubMed based literature, or literature that is more likely to be read by non-chiropractors that figure grows to almost 19 times. If the quality and rate of publication is to improve it is imperative that this imbalance is addressed. Of concern is the fact that publication rates appear to have dropped recently. As a function of all academics, there has been a reduction of 24%, for chiropractic academics there has been a drop of 22%, for non-chiropractic academics there has been a 27% decrease, for doctoral academics there has been a drop of 23%, for non doctoral academics there has been a drop of 10%, and for chiropractic academics with and without doctoral qualifications there has been a drop of 21% and 27% respectively. These numbers are based on already low career average publication rates.

At a time when proponents of EBP suggest more publications and research should be occurring, this performance is disappointing and becomes a concern. Critical appraisal skills of academics are said to be greater than those of new graduates and such skills with new graduates greater than older graduates. There is an implicit requirement to pass on relevant information in the form of teaching to the new graduates by the academics and from the academics to the field practitioner by way of publication [21]. Our research suggests this is not being reflected to the extent that is often quoted in the literature (see Figures 1, 2, 3, 4). As with all tertiary faculties there is an ethical, moral and tacit obligation on the part of chiropractic academia to conduct and publish high quality research. Such research plays an important role in directing educational curricula and ultimately the critical reasoning skills imparted to the student body. This process plays an integral role in developing and fostering the necessary critical reasoning processes required for the future graduate to meet the evolving EBP paradigm present in the public health arena. This is essential if chiropractic is to remain a relevant and competent professional body charged with the role of servicing the greater public good. Otherwise, it is likely that poorly informed and skilled chiropractic students will maintain their disadvantage in the absence of training in an academic culture that promotes the incorporation of evidence and critical thinking skills.

As a whole, the academics within the profession would appear on the basis of the results to have generally not embraced this fact. Such a finding has strong implications for the profession, the schools and the graduating chiropractors. These results may also have many causes: some of which include not having a background that would test their critical reasoning and science based skills, such as a PhD, not gravitating towards inter-professional environments where research has been regarded as a quality highly sought after or thought of as an important component of clinical work or simply not practicing the art and skill of developing, reasoning and implementing experimental protocols aimed at increasing their respective research quanta.

Additional concern stems from the fact that the majority of the research output seems to be occurring from a small number of individuals. Sixty-nine per-cent of total PubMed listed publications have been produced by five academics, 75% of ICL listed publications by six academics and 70% of all recent (five year) publications by four academics. Only three academics have increased their research output in the last five years when compared to their career averages. Based on these statistics, a research paradigm has obviously not been adequately developed within the universities and would appear to be to the detriment of the profession as a whole. This scenario requires immediate and far-reaching redress by the professions own academics, the schools and the political bodies that 'support' the profession. In essence, academics need to be supported and encouraged into acquiring higher degrees so that they in turn may be appropriate role models for those that follow.

Importantly, all academics should be involved in producing research not just with a few individuals. Equally, the profession cannot rely upon non-academics and clinicians to produce the chiropractic research and literature. The failure of the majority of chiropractic program faculty to publish suitably in the fields in which they teach, or at all has been noted previously [1], yet the problem remains unaddressed. Whilst research usually is the province of the academic with or acquiring a PhD, not much is said of the type of study being conducted by the researcher. Interestingly, from an anecdotal point of view, many of the studies appear to be diagnostically relevant investigations, others are investigating the methods used by chiropractors, but very little academic productivity underpins the philosophical basis of the profession's hypotheses despite the fact that these concepts are often presented by the profession as time tested and established. This further highlights the point that teachings within the curricula should be underpinned by solid, well constructed and sound research in the area of the teachings whenever possible, and where such research does not exist, a clear focus on establishing such support should be constructed for the future [22].

### Study limitations

Firstly, despite attempts to obtain detailed and correct information, a possibility exists that the information was not completely timely and as such, any omission of information was a limitation of the study. Secondly, it was not possible to differentiate the quality of, or the significance of the respective publications produced and some publications were not related to chiropractic [23,24]. Thirdly, whilst publication could be interpreted as the ultimate goal of research, its importance must be tempered by the many other factors that drive and promote research and the other forms utilized to disseminate research results. For example we were not able to produce a systematic and objective method of compiling factors such as access to research grants, conference (podium and poster) presentations and higher degree students graduating. However, access to research grants is no guarantee of research quality or publication of results. Fourthly, we did not include chiropractic academics employed at non-chiropractic departments of institutions within Australia (that was not the focus of this paper). There are several individuals teaching in medical and other programs that may score highly in the above analysis. In addition, non-current academics and prominent researchers not listed on the university websites were not included in the review. We reason that these individuals not associated with the university programs were not likely to have the same influence on the student body and hence their influence on the profession may be less than that of the chiropractic academics, even if they are relatively high profile individuals.

Moreover, the total number of publications was calculated for individual authors but represents works of collaboration as well. Due to the collaborative nature of many of the works, an over importance of emphasis may have been placed on prolific authors based on the number of collaborative publications. Additionally, publications are not limited to data papers. Publications include all types of research including reviews and level four case histories. Clearly these publications would be rated differently to a randomized controlled trial, systematic review or large epidemiological study.

We investigated journal publications of academics as the main measure of research output because of the inability to reliably determine all the poster and podium presentations as they often lack a clearly definable and less stringent peer review process. Recent research suggests that only 42% of posters eventually come to publication [25]. In this cohort of British urologists, publications from poster and paper presentations at conferences were similar. However, publications rates are often misrepresented and that is ultimately why we utilized peer reviewed publication rates as an objective measure of academic output [26].

Further limitations exist in that we were not able to differentiate between full-time and part-time academics and we were unable to document the length of time spent as an academic. Part-time academics not listed on the respective websites were also not included and publications appearing outside of the selected databases were not calculated. It should be noted that University A had less full timers (and possibly more fractional staff in compensation), so it may appear that the publication rate is somewhat higher as a function of the mix of staff. University C was the opposite. Thus, it may be that the make up of the full time part time staff ratios had an affect on publication rates.

Additionally, the place of publication is not notable within the analysis. The current analysis does not take into account movement of academics between the universities. One key academic who is listed at one of the universities has accrued the majority of research output at a former university yet the new place of employment could potentially benefit from this assessment of activity. Lastly, using the year of the first published article in producing a publication rate is somewhat arbitrary. This process tells nothing of whether the publications were skewed toward the early, mid or later stages of their respective careers. This omission could have implications for those who are active now when the greatest need for change appears to be upon the profession.

As this analysis attempts to review the research output of the chiropractic academics as a whole, the authors are of the firm belief that the machinations of individuals within the analysis is less important than their overall output.

### Relevance of research productivity to the profession

The analysis of the academic output of the chiropractic academics is important for the dissemination of information to the profession through professional journals and then via teaching that is informed by the content of the publications. This dissemination of information is important within the profession, but it is also important for dissemination to non-chiropractors. This research is most ably done through publication in journals listed in non-chiropractic databases. The mainstream medical literature is generally served by the PubMed database. Any publications appearing in a journal listed in this database potentially receives widespread dissemination to non-chiropractors. Chiropractic academics have advocated publishing chiropractic research in multidisciplinary journals for this reason [3]. Thus the impact of the research is potentially greater than some of the chiropractic journals. It is for this reason that the PubMed database was included in the analysis in this paper.

Some authors have also suggested that the rigor of the PubMed journals is superior to that of non-PubMed journals [2]. Reasons for this position include the greater perceived scrutiny of the journals and the difficulty having manuscripts accepted for publication. However, the only tangible and objective measure of their quality are the journal impact factors. Most chiropractic journals have very low or absent impact factors when many of the medical journals in related fields have much higher impact factors. For example, *JMPT *has an impact factor of 0.8, *Spine *2.3 and *the New England Journal of Medicine *38.6 [27].

It is fair to say that a lack of appreciation for a research culture within the chiropractic profession exists. Much has been spoken about the lack of research in the profession [1]. However, there has been little discussion of the method to correct this perceived deficiency. Much of the discussion has centered on general concepts of recognition and with the role that the individual chiropractor, the local association or granting bodies should play in the remediation of this problem. In this discussion, very little attention has been given to the role of the academics and the universities in creating the desired outcomes.

Upon analysis of the academic output reflected in this paper, it is hard to avoid the grave conclusion that current academics largely appear to be under-equipped to promote the desired outcomes. Large and restricting teaching loads are known to be associated with reduced research output [28]. Whilst it is apparent that many academics have large teaching and administrative roles within their departments, such commitments should not absolve them from the primary role of producing research within research based institutions. This defense is often cited as the reason for the lack of research productivity and it has merit for some academics that have been engaged primarily as educators. However, the poor publication rates produced by chiropractic academics in comparison with the rest of the university academics weakens this argument [16], as it is likely that teaching and administrative pressures are uniform across the university and common for all academics regardless of discipline. Regardless, the majority of academics have general academic appointments that require a balanced approach to teaching, administration and research, the three pillars of academic life.

Important in any deliberation of the available time to produce research or the requisite training to do so is the knowledge that if academics see themselves and are celebrated by their peers as researchers they are invariably more productive. In a survey of clinical psychologists in California academics that saw themselves as researchers rather than teachers had a higher publication rate despite other variables such as grants, time in research and type of institution attended [29]. By contrast, research by Hickling [30] reported that academics with increased teaching loads and increased clinical commitments have been associated with increased claims of performing research (research in progress) and a reduced research publication rate. The authors of this paper contend that similar problems have been observed in chiropractic academics in Australia.

If academics have strayed into a 'teaching only' realm of existence they have done so by choice or perhaps at the behest of more senior departmental staff. The second more realistic likelihood would appear that departmental research funding has become squeezed in recent years or is proffered away into side projects such as distance education programs or other post graduate fee-paying programs. These programs all require significant lead-time in development that does not usually come from the core business of teaching, but might come from the softer option of research. Thus, the chiropractic academic culture appears to be heading down a slippery road of promoting short-term revenue raising teaching options at the expense of the longer-term, and professionally healthier alternative, research activity, in any case, whatever the individual university pressures may be this ominous trend must be redressed sooner rater than later.

Interestingly, research productivity has been associated with a positive self-image as a researcher. Research has found that: spending more than 25% of time pursuing research, receiving specialist training, having employment in a university or government institution and a possessing a good degree of self motivation to produce research are characteristics of a successful researcher [31,32]. Effective research mentoring is also crucial to establishing a research culture [33]. In support of this contention, it has been shown that high publishing academics are significantly more likely to produce high publishing doctoral graduates [18]. This suggests that academics who have embraced a research culture are likely to impart this on their students. Thus, it is our belief that a positive research culture needs to be engrained into the chiropractic student body at the university level. This needs to begin with research training units being taken seriously by faculty staff and students. Units need to be constructed that are relevant and interesting (difficult but not impossible to do) and not simply being viewed as an activity subject that is irrelevant in the world of hands-on chiropractic. Making the teaching of research interesting and relevant to chiropractic students remains the primary challenge of the academic. In addition, critical analysis skills need to be fostered early and continuously throughout the education of the chiropractor. Research and critical reasoning skills must be taught and reinforced throughout the entire chiropractic program, embedded into all the units offered not just marginalized to research methodology training and dismissed. To achieve this goal, additional time needs to be devoted to research training of student chiropractors (at all levels). Students need to understand and be exposed to the benefits of being a 'scientific clinician' in this era of EBP, rather than the almost continual negative side of the EBP push. They and the academics need to understand that a balance is required of the science, philosophy and art of chiropractic, as arguably no such balance apparently exists at the moment. Students must be encouraged to understand that a clinical career that is balanced with the science, art and philosophy of modern day chiropractic is in fact feasible, worthwhile and highly sought after. The 'representative' professional bodies within Australia must then further extend this message by espousing these virtues to be of benefit to the profession, the public good as well as the individual.

We also suggest that whilst not abandoning pure science research, there needs to be a change of research focus into clinically relevant outcomes based research. The 21^st ^Century is the era of EBP and chiropractic must accept and embrace this: failure to do so is at the expense of its own long-term future. Mechanistic type research is of little use when the intervention employed fails to withstand the scrutiny of rudimentary scientific rigor or has not yet been shown to be clinically effective, relevant, or equal to a placebo intervention.

In addressing this problem, faculty members should be encouraged into a pathway of higher academic research based degrees and they should likewise encourage graduates of the programs, particularly for high achieving students to follow a similar path. This should address publication rates as doctoral candidates have been shown to have a high publication rate [4] and doctoral graduates who become faculty members have a significantly increased chance of producing a high publication rate than those without a doctoral degree [18]. The offering of scholarships and other grants would act as a suitable incentive. Research in an Australian university medical setting has shown that publication rates improve with university support [34,35] and when there is a greater emphasis on scholarly activity [36]. The overcoming of barriers to research productivity requires the coordinated support of the academics, the universities and the profession [32].

Supervision of chiropractic academics is also an issue. As there are so few academics with the requisite qualifications to supervise research students, it is difficult to supervise an entire staff wanting to acquire research training. Collaborations between departments need to be explored and fostered by the respective university hierarchies. Thus, these teaching institutions must lead the way in order to facilitate this process within timely manner and with the appropriate talent and vital long term seed funding. They should allow the entrance of faculty into higher degree (MSc, MPhil and PhD) programs as a special sub category with a window of sufficient grace for a period that would adequately expedite such a program. In that time, a special program of training could be put in place to bring all staff into a more appropriate research frame of thought. Such remediation is sorely needed as research conducted by supervisors who only have equivalent qualifications as their students is an obvious and unacceptable situation and one which would not be tolerated in any university setting.

Additionally, utilizing the small number of research degree graduates in the teaching of chiropractic programs is a must. These individuals are research trained chiropractors usually with significant clinical experience who are more likely to produce publications [18]. They would make ideal candidates for post doctoral positions and remain role models for a developing profession, and a useful vehicle for the departmental administrators to actively engage sessional academics who have an interest in research, as opposed to the average sessional academic who may not, and is only engaged to teach. The admission of such appropriately qualified and motivated sessional academics will only help to strengthen chiropractic departments within universities and the role of the profession within the wider community.

The dilemma lies in that the research culture required to execute these changes is not apparent within the majority of faculty members including some department heads. Whilst much is said of the need for research by influential members of the chiropractic academic community [2,3], the collective research output of the universities (under their tenure) has not, to date, reflected the veracity of their writings. Successful research based academics have largely been self motivated individuals who have excelled in spite of the burdening systems they work under.

The authors contend that it is difficult to teach critical analysis skills and appreciation for research when it was not properly taught at most, if not at all within chiropractic schools, and unless the individual has chosen to acquire research skills independently of their chiropractic education, they are probably insufficiently prepared for today's evidence-based era. This has largely resulted in the creation of faculty environments where emerging interests in well thought out and conducted research has competed for revenue earning endeavours leading to an urgent requirement to train the trainers. Chiropractic education planners need to make the possession of a research degree by all faculty teaching in chiropractic programs an indispensable priority. Current staff should be strongly encouraged that minimum publication and research targets need to be established and achieved. They should be supported to do so with innovative programs which reduce the time impost from teaching and increase funding from departments, the profession and relevant granting bodies. Even with such support, chiropractic faculty need to accept the fact that they have a responsibility as academic professionals to teach best practice, and best practice is underpinned by research. The authors believe that this is a reasonable expectation for the profession and the public to hold.

One method to measure the collective output of the profession would be to develop a central chiropractic research database to monitor the progress of research in the profession, that would run much along the lines of the Australian Physiotherapy PEDro model [37]. Such a site could be web based and have open access and could help promote more competitive and productive outcome through the fostering of a single, coherent spirit of chiropractic advancement. This could also facilitate the progressive professional culling of less active faculty staff: such a 'publish or perish' mentality has been suggested as a healthy and refreshing change [1], and one that already exists in other fields [38]. Also, appointment of future faculty will need to heavily favor those with an active research and academic record, or have an ability to acquire such a record.

The significance of the situation is that an inability of graduating chiropractors to attain sufficient critical analysis skills and an appreciation for research leaves them open to suspect practice management schemes and other entrepreneurs preying on the profession [9]. The chiropractic profession has been all too often singled out for making treatment claims about care that go well beyond the limits of supportive data, whereas other professions seem not to suffer from this insidious dis-ease [7]. The rise of mercantilism, for some, by the placement of patients' needs second to the commercial interests of the chiropractor has been a shock to a profession with apparent lofty healthcare goals [39]. Inclusive of this is the almost repeated sales pitches for invalidated, un-measurable and unreliable outcome measures. Such examples of these unscrupulous methods include: surface EMG equipment, shopping centre spinal screens, routine and repeat full spinal X-ray services, the 'latest' technique seminars and advertising which promises to "double your practice" that either lack supportive evidence or have the evidence overstated by pseudo-scientists and chiro-evangelists to pander to the egotistical and financial concerns of some chiropractors. The inappropriate treatment and billing practices adopted by some members of the chiropractic profession that often place the economic interests of these chiropractors before the best interests of the patient has already been identified as a significant issue affecting the credibility of the profession in the immediate and near future [40]. The almost continual stream of complaints made against high volume, pre-paid contract chiropractors at the Registration Board level would support this viewpoint. The end result is that this treatment undermines public trust in all members of the chiropractic profession, whether they are participants in the practice or not [40]. The chiropractic profession, particularly the easily influenced newer graduate needs to retain the ability to remain both openly and vocally skeptical to such external encouragements that result in the unethical treatment of the Australian public. The consequence's of this is that a lack of professional credibility for chiropractic hastens the profession's marginalization and/or exclusion because of a systemic failure to evolve with EBP in the health science professions. It also makes chiropractic's call for greater equity through government and national based funding schemes and greater integration and inter-professional cooperation feeble and non-sensible.

## Conclusion

Australian university based chiropractic academics on average demonstrate a poor research culture when using peer reviewed research publication as a measure of output. Of concern is the recent trend for a falling publication rate at a time of increasing reliance on research to inform practice. This lack of research output may have significant impact on the attitudes held by chiropractic graduates toward the importance of research training including critical reasoning skills required to effectively select clinical outcomes supported by research rather than hype. A challenge remains for the academics, the institutions and the profession to place remedial steps in place to arrest and reverse this decline in research output. It is likely that a coordinated approached from these groups will be required to facilitate this change.

## Competing interests

No funding was received in the preparation of this manuscript. JR has no conflict of interest directly related to the content of the manuscript, WH and AV are PhD candidates at Macquarie University, HP is a part-time academic at Macquarie University and RB is a full-time academic at Macquarie University. The authors were not involved in collecting the data about their own publications.

## Authors' contributions

WH conceived of the study, participated in its design, constructed the literature review and helped to draft and edit the manuscript.

HP conceived of the study, participated in its design, constructed the literature review and helped to draft and edit the manuscript.

JR participated in its design, constructed the literature review and helped to draft and edit the manuscript.

AV participated in its design, constructed the literature review and helped to draft and edit the manuscript.

RB participated in its design, constructed the literature review and helped to draft and edit the manuscript.

All authors read and approved the manuscript.
